# Work participation after receiving multidisciplinary treatment or acceptance and commitment therapy intervention for return to work: long-term follow-up of a randomized controlled trial among sick-listed individuals with mental disorders and/or chronic pain

**DOI:** 10.1186/s12889-024-21116-1

**Published:** 2024-12-21

**Authors:** Erik Berglund, Ingrid Anderzén, Magnus Helgesson, Per Lytsy, Åsa Andersén

**Affiliations:** 1 Department of Public Health and Caring Sciences, Uppsala University, Box 564, Uppsala, SE-751 22 Sweden; 2 Division of Insurance Medicine, Department of Clinical Neuroscience, Karolinska Institutet, Stockholm, SE-171 77 Sweden

**Keywords:** Return to work, Vocational rehabilitation, Multidisciplinary rehabilitation, Chronic pain, Mental disorders, Sick leave

## Abstract

**Background:**

The return-to-work (RTW) process for individuals on long-term sick leave can be complex. Vocational rehabilitation may facilitate RTW; however, many intervention studies often have relatively short follow-up periods. The purpose of this study was to assess long-term work participation 2–7 years after the initiation of a three-armed randomized controlled trial aimed at RTW for individuals on long-term sick leave because of mental disorders and/or chronic pain.

**Methods:**

This study followed 220 participants out of 402 (response rate 55%, 205 female) who had previously participated in a randomized controlled trial. They were allocated to one of three groups: multidisciplinary team assessment and individualized treatment (MDT), acceptance and commitment therapy (ACT) or a control group. The participants were followed up at two, three, four, five, six, and seven years after inclusion. The outcome, work participation, was assessed using registry data and defined as having the main source of annual income from paid work during the follow-up years.

**Results:**

Participants in the MDT intervention group were, to a larger extent, in paid work during follow-up in years four (9.0% points), five (2.5% points), six (7.6% points), and seven (4.1% points) after inclusion, compared to the control group. Participants in the ACT intervention group were, to a larger extent, in paid work during follow-up in years four (14.8% points), six (17.6% points), and seven (13.9% points) after inclusion, compared to the control group.

**Conclusion:**

This study, primarily involving female individuals on long-term sick leave, suggests that both MDT and ACT interventions can improve long-term work participation. The results also indicate some time-lag effect of the interventions.

**Trial registration:**

The original randomized study was registered at the Clinicaltrials.gov Register Platform (ID NCT03343457); registered on November 15, 2017 (retrospectively registered).

## Background

The costs and burdens of long-term sick leave are substantial for individuals, employers, and societies [1, 2]. Common mental disorders and chronic pain are the two most frequent reasons for long-term sick leave in many countries, including Sweden [3, 4]. Besides the individual suffering related to mental disorders and chronic pain, there are significant societal costs related to productivity losses [5]. People on long-term sick leave often face complex challenges when returning to work [6], and longer periods of sick leave are known risk factors for failure to return-to-work (RTW) [7, 8]. Other factors associated with poorer RTW outcomes include older age, female gender, pain, disability, depression, high work demands, previous sick leave, unemployment, and activity limitations [9].

Several strategies have been proposed to prevent work absence and facilitate RTW, such as unimodal or multimodal intervention programs (team-based assessments and rehabilitation efforts by multiple health professionals) [10, 11], and interventions that target a structural level [12]. Systematic reviews have found that multimodal interventions and those including workplace involvement can reduce work absence and improve RTW rates in individuals with pain and mental health conditions [13–17]. Studies have also highlighted the importance of workers’ attitudes and emotions toward RTW [18, 19]. Similarly, qualitative studies suggest that RTW should be considered as a multi-stage process, which entails changes in workers’ thoughts, feelings, and behaviors over time [20, 21].

In 2008/2009, the social insurance system in Sweden was reformed, and a time limit was introduced for obtaining long-term sickness benefits; moreover, people on sick leave were transferred to the Swedish Public Employment Service (SPES) to assess their work ability [22]. Between 2010 and 2012, an intervention project was implemented, targeting individuals on long-term sick leave who were at risk of losing their compensation from the Swedish Social Insurance Agency (SSIA). The short-term effects of the interventions, measured during a one-year follow-up, showed that the multidisciplinary team assessment and individualized treatment intervention increased RTW rates [23] and improved self-rated employability [24]. The sole ACT intervention was also found to increase self-rated employability [24]. There is a general lack of long-term follow-up studies on RTW interventions, as these studies face challenges due to factors such as changing work conditions and health over time. This study aimed to follow up on a three-arm intervention study for RTW [23–25], carried out between 2010 and 2012, with individuals on long-term sick leave or temporary disability pension due to mental disorders and/or chronic pain. The objective was to assess and demonstrate work participation patterns following a multidisciplinary team assessment and individualized treatment (MDT) and unimodal psychological treatment with acceptance and commitment therapy (ACT) intervention during a two to seven-year follow-up.

## Methods

The interventions were conducted within a randomized controlled trial (RCT), implemented in two phases from 2010 to 2012. In the first phase, female participants were randomly block-allocated in triplets to either: (1) Multidisciplinary team assessment and individualized treatment (MDT), (2) Acceptance and commitment therapy (ACT), or (3) a control group. In the second phase of the intervention, two-thirds of the participants were randomized to the MDT group and one-third to the control group. Both female and male participants were included in this phase. This long-term follow-up study evaluates pooled data from both phases. Parts of the questionnaire-based data used in the present study have been used in previous studies [23–26].

### Participants and inclusion

Participants eligible for the study included women and men (men were included only in the second phase) who were on part- or full-time sick leave or receiving a temporary disability pension due to a mental disorder and/or a pain-related diagnosis in Uppsala County, Sweden. Details regarding participant identification, selection, inclusion and exclusion criteria, and the allocation procedure for the RCT have been published previously [24]. Four hundred and forty-eight (448) men and women gave their informed consent to participate in the study. In total, 34 participants were excluded for various reasons: not meeting the inclusion criteria, fulfilling the exclusion criteria, declining to participate, or due to ethical considerations. The SSIA randomly allocated 414 individuals into one of the following groups: the MDT group (*n* = 172), the ACT group (*n* = 101), or the control group (*n* = 141). The sole ACT intervention group was removed after the first phase of the project; thus, fewer participants were allocated to the ACT group compared to the MDT and the control group. The reason for removing the sole ACT intervention group was that during phase 1, some participants randomized to the ACT group expressed a lack of interest in receiving ACT treatment.

**Fig. 1:**
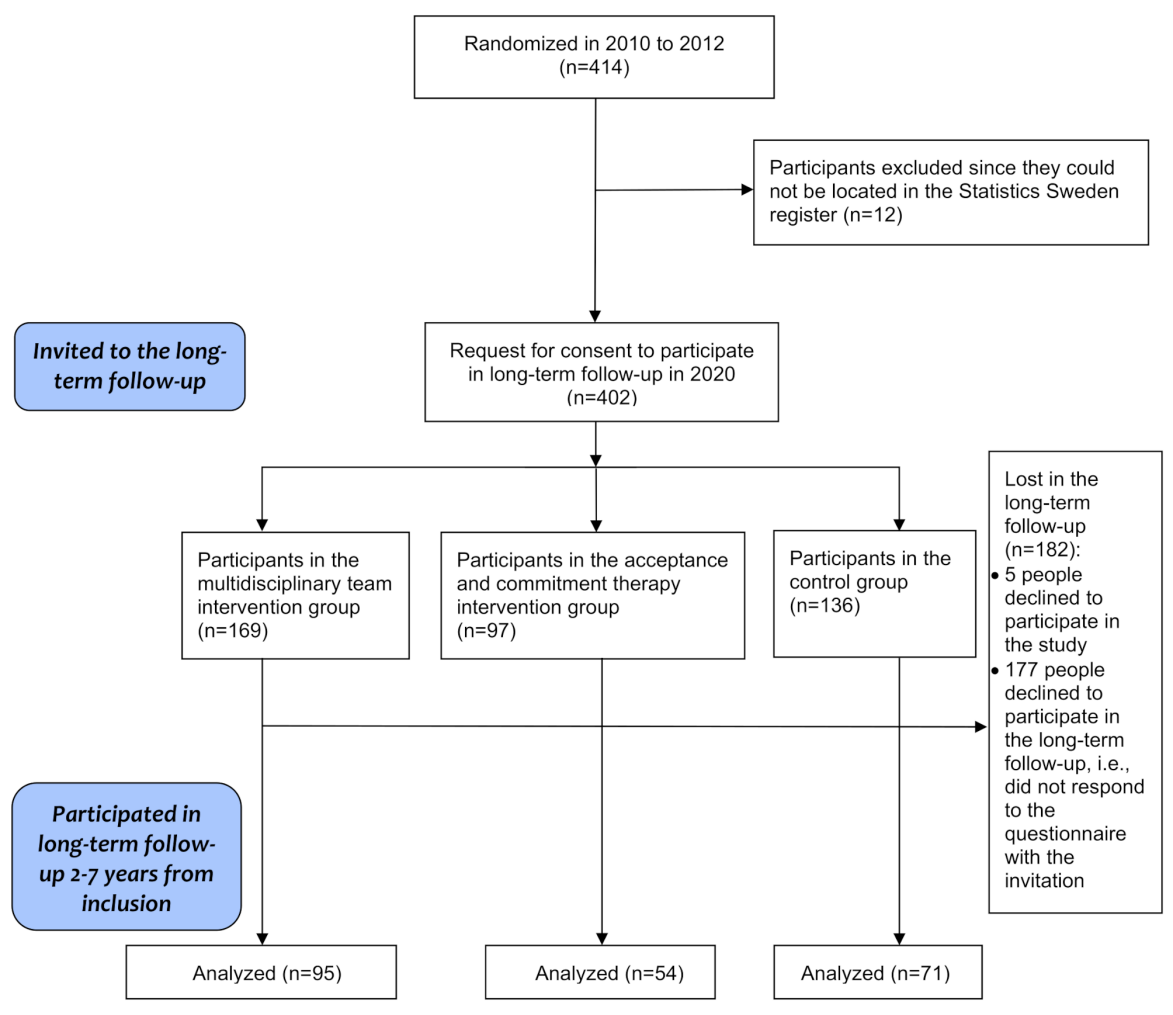
Flow chart of the long-term follow-up

In 2020, a request for consent to participate in a long-term follow-up, along with a questionnaire, was distributed to 402 study participants identified through Statistics Sweden’s register. Twelve participants could not be located in the Statistics Sweden register and were excluded. Two hundred and twenty (220) participants responded to the questionnaire and provided consent to collect registry data regarding their income and/or activity during the follow-up period (see Fig. 1). The overall response rate for the long-term follow-up request was 55% (220/402). Response rates for the long-term follow-up were similar across the randomized groups.

### Interventions and control groups

Participants randomized to the MDT group met individually with professionals from a multidisciplinary team, including a psychologist, a physician, an occupational therapist, and a social worker. Each professional assessed the participant’s strengths and limitations for RTW from their perspective and established an individualized rehabilitation plan in collaboration. Based on the individual assessment, suggested interventions could include further medical investigation/treatment, occupational evaluation and training, social and economic counseling and psychological treatment using ACT, provided by a psychologist. ACT is a form of cognitive behavioral therapy (CBT) that uses acceptance and behavioral strategies to increase psychological flexibility and functioning rather than aiming to reduce symptoms [27, 28]. Participants allocated to the ACT group received solely ACT. The ACT intervention involved assessment and treatment conducted by a psychologist; if a participant could not come to the clinic due to pain or other disabilities, sessions were offered outside of the clinic setting. Adherence to the planned interventions and the number of meetings completed varied among participants.

All participants randomized to the intervention groups received structured collaboration between the SSIA and the SPES. A contact person from either the multidisciplinary team or a psychologist for the ACT group participated in the meetings with representatives of the SSIA and the SPES, together with the participant. The main purpose of this organizational cooperation was to increase the participants’ engagement in the rehabilitation process and establish agreements on the RTW goals for both the participants and the participating organizations.

The control group did not receive any intervention organized through the intervention project and followed the standard procedures when transitioning from the SSIA to the SPES.

### Questionnaire to participants before the intervention

Data collected before the intervention (pre-treatment) included demographic data such as the respondent’s gender, age, and educational level (categorized as compulsory school, secondary school or equivalent, or university). A member of the research group classified the diagnosis stated on the sick leave certificates as any of the following: pain, mental disorders, or a combination of both. Anxiety and depression levels were assessed using the Hospital Anxiety and Depression Scale (HADS) [29]. The General Self-Efficacy Scale (GSE) was used to measure the participants’ belief in their ability to handle various difficult demands in life [30]. A mean GSE value was calculated as the sum of all answers divided by the number of statements, provided that no more than three statements were missing [31]. The current version of the GSE has been translated into Swedish and validated [32]. The work-related variables used in this study included employment contract status (unemployed or employed), the extent of sick leave (full-time or part-time), and number of years with income replacement, which were collected through the SSIA registry data.

### Outcome measures

To measure long-term work participation, participants were followed up using data from the Longitudinal Integration Database for Health Insurance and Labor Market Studies (LISA) register compiled by Statistics Sweden. LISA provides information on annual income from different sources and activities, such as employment, student allowances, parental benefits/benefits for the care of a closely related person, sickness benefits, unemployment benefits, permanent sickness compensation/disability benefits, social allowances, labor market training or employment programs, and old-age pensions. In addition, LISA also classifies individuals with no registered income.

The outcome measure uses an individual’s main source of registered income or activity in a follow-up year, counted the year after inclusion. Data from 2012 to 2018 were used to categorize individuals based on their main source of income during follow-up in years two, three, four, five, six, and seven. The starting point for this categorization was the main source of income for a specific year, as classified by Statistics Sweden. Specifically, it was determined by the source of income that provided at least 50% of an individual’s total annual income that year. According to this classification, each individual was assigned to only one of the following categories with the main source of income coming from: (1) employment (including self-employment), (2) student allowance, (3) parental benefits/benefits for care of a closely related person, (4) sickness benefits, (5) unemployment benefits, (6) permanent sickness compensation/disability benefits, (7) social allowances, (8) labor market training or employment program, (9) old-age pension, or (10) unknown/no registered income. This variable has been used previously in longitudinal studies regarding sick leave [33, 34]. The outcome variable was further dichotomized into those having a main source of income from work and those who did not, for the binary logistic regression analyses. Having a main source of income from employment was categorized as being in work during a particular follow-up year. Having a main source of income from student allowances, sickness benefits, unemployment benefits, permanent sickness compensation/disability benefits, social allowances, labor market training or employment program, or having no registered income was categorized as not participating in work during a particular follow-up year. Individuals receiving parental benefits/benefits for care of a closely related person or old-age pensions were excluded from the binary logistic regression analysis for the particular year that such income was the main source of income.

### Analyses

Differences in the outcomes between the intervention and the control groups were demonstrated with descriptive statistics and graphs for each follow-up year. The data were analyzed according to the intention-to-treat (ITT) principle. Binary logistic regression models were used to investigate differences in work participation outcomes between each intervention group and the control group. Both crude and adjusted analyses were carried out. In the adjusted models, the effect of group allocation (MDT, ACT vs. control group) was adjusted for age, education level, sick leave diagnosis, HADS, GSE, employment contract, the extent of sick leave, and years with sick leave compensation. The main analyses used data from respondents who were followed up in the LISA registry. Due to a large number of non-responders to the long-term follow-up questionnaire and the subsequent missing values in the outcome variable (45%), a sensitivity analysis was also performed, in which worst-case scenario analyses were carried out [35]. In the sensitivity analysis, non-responders (*n* = 182) were assumed not to have their main income from employment (reference category). Dropout analyses were also performed by comparing the pre-treatment questionnaire data between those who participated and those who dropped out of the long-term follow-up. Chi-square tests were used to compare differences in distributions, and the Mann-Whitney U test was used to compare differences in median values in the dropout analyses. All tests were two-sided, and a *p*-value of < 0.05 was considered statistically significant. Nagelkerke’s R squared (r^2^) was calculated to describe the goodness of fit for the adjusted logistic regression models. The statistical analyses were performed using SPSS statistics (IBM Corp, Armonk, New York), version 27.0.

### Ethical considerations and trial registration

The Regional Ethical Review Board of Uppsala approved the initial intervention study on April 8, 2010 (Reg. no. 2010/088) following the Declaration of Helsinki. The long-term follow-up study was approved on April 12, 2020. The study was registered at the Clinicaltrials.gov Register Platform (ID NCT03343457) on 15/11/2017 (retrospectively registered). All participants provided written informed consent to participate in the intervention study. By returning the questionnaire sent out in 2020, participants were deemed to have consented to participate in the long-term follow-up study.

## Results

### Pre-treatment data and dropout analysis

The pre-treatment characteristics of the participants who consented to the long-term follow-up are presented in Table 1. The average age of participants was 49.4 years (SD 7.8). The study group was predominantly female, with 93.2% women and 6.8% men. Most participants had completed secondary school or equivalent education, with university education being the most common.

**Table 1:** Pre-treatment characteristics of study participants who consented to participate in the long-term follow-up, distributed by group allocation

		MDT^a^ group *n* = 95 (43.2)	ACT^b^ group *n* = 54 (24.5)	Control group *n* = 71 (32.3)	Total *n* = 220 (100)
Sex	Female, n (%)	86 (90.5)	54 (100)	65 (91.5)	205 (93.2)
	Male, n (%)	9 (9.5)	0 (0.0)	6 (8,5)	15 (6.8)
Age, years	Median (md), mean (SD)	50, 50.2 (7.6)	49, 49.2 (8.0)	50, 48,4 (7.9)	50, 49.4 (7.8)
Education level	Compulsory school, n (%)	14 (17.3)	3 (6.8)	10 (16.9)	27 (14.7)
	Secondary school or equivalent and university education, n (%)	67 (82.7)	41 (93.2)	49 (83.1)	157 (85.3)
Sick leave diagnosis	Pain diagnoses	43 (45.3)	19 (35.2)	32 (45.1)	94 (42.7)
	Mental disorders diagnoses	26 (27.4)	22 (40.7)	20 (28.2)	68 (30.9)
	Both pain and mental disorder diagnoses	26 (27.4)	13 (24.1)	19 (26.8)	58 (26.4)
Hospital Anxiety and Depression Scale (HADS)	Anxiety, md, mean (SD)	10, 9.8 (4.7)	9, 9.7 (4.9)	11, 10.5 (4.6)	10, 10.0 (4.7)
	Depression, md, mean (SD)	9, 8.9 (5.0)	8, 8.0 (4.3)	8, 7.9 (3.8)	8, 8.4 (4.5)
General self-efficacy (GSE)	GSE, md, mean (SD	2.5, 2.4 (0.7)	2.4, 2.5 (0.6)	2.3, 2.4 (0.6)	2.4, 2.4 (0.6)
Employment contract	Employed, n (%)	70 (73.7)	36 (66.7)	44 (62.0)	150 (68.2)
	Not employed, n (%)	25 (26.3)	18 (33.3)	27 (38.0)	70 (31.8)
Extent of sick leave	Part-time, n (%)	52 (55.9)	28 (51.9)	39 (55.7)	119 (54.8)
	Full-time, n (%)	41 (44.1)	26 (48.1)	31 (44.3)	98 (45.2)
Years with sick leave compensation	Md, Mean (SD)	9, 8.3 (3.5)	7, 7.1 (3.1)	8, 8.1 (3.0)	8, 7.9 (3.3)

Figures as percentages, if not otherwise stated

^a^Multidisciplinary team assessment and individualized treatment (MDT) intervention group

^b^Acceptance and commitment therapy (ACT) intervention group

At the time of inclusion, 42.7% of the participants were on sick leave for pain-related conditions, 30.9% for mental disorders, and 26.4% for a combination of mental disorders and pain-related conditions. Most participants (68.2%) had an employer at the time of inclusion, and the average time on sick leave or temporary disability pension was 7.9 years (SD 3.3). Of the participants, 45.2% were on full-time sick leave, and 54.8% were on part-time sick leave at inclusion.

Differences between the dropouts and participants fulfilling the long-term follow-up were investigated. Statistically significant differences (*p* < 0.05) were found in the following areas: education level (lower among dropouts), HADS anxiety (higher among dropouts), HADS depression (higher among dropouts), GSE (lower among dropouts), and extent of sick leave (full-time sick leave being more common among dropouts).

### Work participation and main sources of income among participants at long-term follow-up

Table 2 presents the main sources of income among participants at follow-up in years two, three, four, five, six, and seven in the MDT, ACT, and control groups. Two years after inclusion in the intervention study, 47.4% of participants in the MDT group and 46.3% in the ACT group had employment as their main source of income. In comparison, 43.7% of participants in the control group had employment as their main source of income two years after inclusion. There were no major differences at follow-up in year three, 45.3% of participants in the MDT group, 50.0% of participants in the ACT group, and 46.5% of the participants in the in the control group had income from employment as their main source of income. At follow-up in year four, 44.2% of participants in the MDT group, 50.0% in the ACT group, and 35.2% in the control group had employment as their main source of income. At follow-up in year five, 42.1% of participants in the MDT group, 40.7% in the ACT group, and 29.6% in the control group had employment as their main source of income. At follow-up in year six, 40.0% of participants in the MDT group, 50.0% in the ACT group, and 32.4% in the control group had employment as their main source of income. Finally, at follow-up in year seven, 42.1% of participants in the MDT group, 51.9% in the ACT group, and 38.0% in the control group had employment as their main source of income. The proportion of participants receiving old-age pensions increased during the follow-up years. See Table 2; Fig. 2a-c.

**Table 2:** Main sources of income and activities among participants in the MDT, ACT, and control groups at follow-up in years two, three, four, five, six, and seven after inclusion

	Two years	Three years	Four years	Five years	Six years	Seven years
**Multidisciplinary treatment group**						
Employment	45 (47.4)	43 (45.3)	42 (44.2)	40 (42.1)	38 (40.0)	40 (42.1)
Student allowance recipients	0 (0)	0 (0)	0 (0)	0 (0)	0 (0)	0 (0)
Parental benefits/Benefits for care of a closely related person	0 (0)	0 (0)	1 (1.1)	1 (1.1)	1 (1.1)	0 (0)
Sickness benefits	16 (16.8)	16 (16.8)	11 (11.6)	8 (8.4)	4 (4.2)	3 (3.2)
Unemployment benefits	0 (0)	1 (1.1)	0 (0)	1 (1.1)	1 (1.1)	1 (1.1)
Permanent sickness compensation/Disability benefits	12 (12.6)	20 (21.1)	24 (25.3)	28 (29.5)	28 (29.5)	28 (29.5)
Social allowances	1 (1.1)	1 (1.1)	1 (1.1)	1 (1.1)	1 (1.1)	1 (1.1)
In labor market training or employment program	14 (14.7)	4 (4.2)	4 (4.2)	2 (2.1)	3 (3.2)	2 (2.1)
Old-age pensions	6 (6.3)	10 (10.5)	12 (12.6)	14 (14.7)	19 (20.0)	19 (20.0)
Data on income are missing in the registry	1 (1.1)	0 (0)	0 (0)	0 (0)	0 (0)	1 (1.1)
**ACT group**						
Employment	25 (46.3)	27 (50.0)	27 (50.0)	22 (40.7)	27 (50.0)	28 (51.9)
Student allowance recipients	0 (0)	0 (0)	1 (1.9)	2 (3.7)	0 (0)	0 (0)
Parental benefits/Benefits for care of a closely related person	1 (1.9)	0 (0)	0 (0)	0 (0)	0 (0)	1 (1.9)
Sickness benefits	12 (22.2)	11 (20.4)	8 (14.8)	7 (13.0)	3 (5.6)	1 (1.9)
Unemployment benefits	0 (0)	0 (0)	0 (0)	0 (0)	0 (0)	0 (0)
Permanent sickness compensation/Disability benefits	2 (3.7)	6 (11.1)	12 (22.2)	14 (25.9)	12 (22.2)	10 (18.5)
Social allowances	1 (1.9)	2 (3.7)	1 (1.9)	1 (1.9)	2 (3.7)	2 (3.7)
In labor market training or employment program	12 (22.2)	6 (11.1)	2 (3.7)	2 (3.7)	1 (1.9)	1 (1.9)
Old-age pensions	1 (1.9)	2 (3.7)	3 (5.6)	5 (9.3)	8 (14.8)	10 (18.5)
Data on income are missing in the registry	0 (0)	0 (0)	0 (0)	1 (1.9)	1 (1.9)	1 (1.9)
**Control group**						
Employment	31 (43.7)	33 (46.5)	25 (35.2)	21 (29.6)	23 (32.4)	27 (38.0)
Student allowance recipients	1 (1.4)	1 (1.4)	1 (1.4)	2 (2.8)	1 (1.4)	1 (1.4)
Parental benefits/Benefits for care of a closely related person	1 (1.4)	1 (1.4)	0 (0)	0 (0)	0 (0)	0 (0)
Sickness benefits	18 (25.4)	16 (22.5)	21 (29.6)	17 (23.9)	13 (18.3)	9 (12.7)
Unemployment benefits	1 (1.4)	1 (1.4)	1 (1.4)	0 (0)	2 (2.8)	0 (0)
Permanent sickness compensation/Disability benefits	10 (14.1)	13 (18.3)	13 (18.3)	19 (26.8)	21 (29.6)	22 (31.0)
Social allowances	1 (1.4)	1 (1.4)	3 (4.2)	1 (1.4)	2 (2.8)	2 (2.8)
In labor market training or employment program	6 (8.5)	2 (2.8)	2 (2.8)	4 (5.6)	2 (2.8)	1 (1.4)
Old-age pensions	1 (1.4)	2 (2.8)	5 (7)	7 (9.9)	7 (9.9)	9 (12.7)
Data on income are missing in the registry	1 (1.4)	1 (1.4)	0 (0)	0 (0)	0 (0)	0 (0)

Main sources of income and activities among participants at follow-up in years two, three, four, five, six, and seven years after inclusion in the multidisciplinary team assessment and individualized treatment (MDT) group, acceptance and commitment therapy (ACT) group, and the control group

**Fig. 2a:**
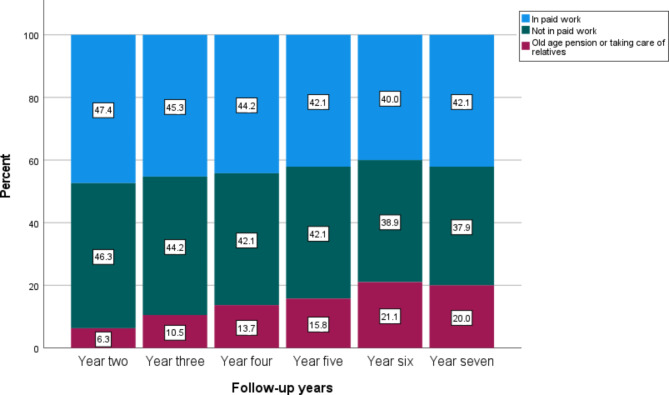
Main source of income among participants in the multidisciplinary team assessment and individualized treatment (MDT) intervention group at follow-up in years two, three, four, five, six, and seven

**Fig. 2b:**
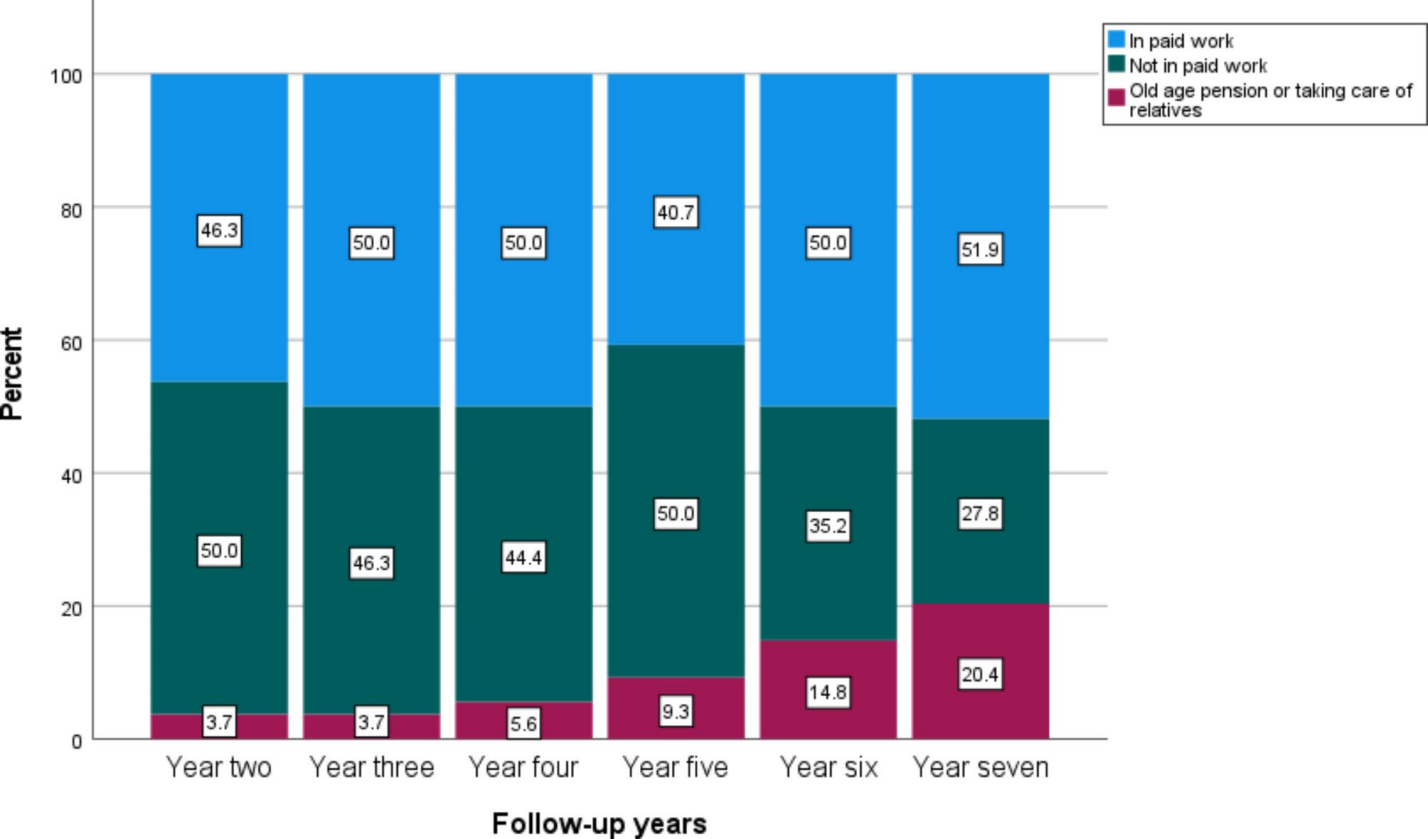
Main source of income among participants in the acceptance and commitment therapy (ACT) intervention group at follow-up in years two, three, four, five, six, and seven

**Fig. 2c:**
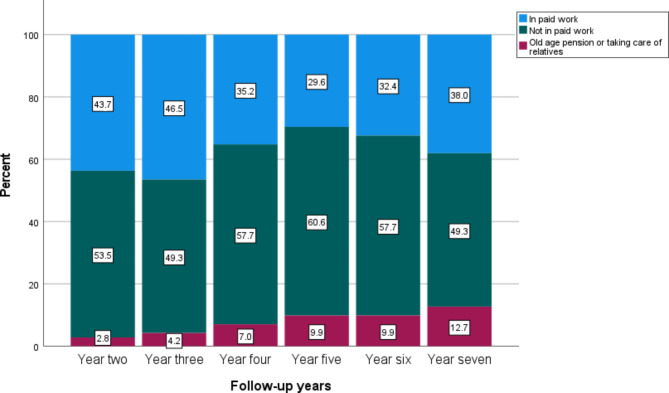
Main source of income among participants in the control group at follow-up in years two, three, four, five, six, and seven

### Logistic regression models

The crude binary logistic regression analysis showed statistically significant differences in the work participation outcome, favoring the MDT group over the control group at follow-up in year five (OR 2.05, 95% CI 1.04–4.05). In the adjusted logistic regression analyses, statistically significant differences were found in the work participation outcome, also favoring the MDT group over the control group at follow-up in years four (adjusted OR 4.14, 95% CI 1.55–11.07), five (adjusted OR 4.80, 95% CI 1.76–13.09), six (adjusted OR 3.94, 95% CI 1.48–10.49), and seven (OR 2.67, 95% CI 1.02–6.98). See Table 3.

**Table 3:** Logistic regression analyses presenting odds ratios for having employment as the main source of income at follow-up in years two, three, four, five, six, and seven

		Two years	Three years	Four years	Five years	Six years	Seven years
		OR (95% CI)	OR (95% CI)	OR (95% CI)	OR (95% CI)	OR (95% CI)	OR (95% CI)
Crude	Group allocation						
	Control	1	1	1	1	1	1
	ACT intervention^a^	1.14 (0.55–2.34)	1.15 (0.56–2.36)	1.85 (0.88–3.87)	1.67 (0.77–3.60)	2.53 (1.16–5.52)	2.42 (1.08–5.40)
	MDT intervention^b^	1.25 (0.67–2.36)	1.09 (0.57–2.06)	1.72 (0.89–3.33)	2.05 (1.04–4.05)	1.83 (0.93–3.62)	1.44 (0.73–2.83)
Adjusted models^d^	Group allocation						
	Control	1	1	1	1	1	1
	ACT intervention^a^	1.28 (0.42–3.94)	1.52 (0.53–4.36)	3.21 (1.03–9.96)	1.87 (0.63–5.55)	3.73 (1.20-11.59)	3.99 (1.23–12.91)
	MDT intervention^b^	1.90 (0.72–5.03)	1.86 (0.76–4.56)	4.14 (1.55–11.07)	4.80 (1.76–13.09)	3.94 (1.48–10.49)	2.67 (1.02–6.98)
	Age	1.06 (0.99–1.12)	1.02 (0.96–1.08)	0.99 (0.93–1.06)	1.00 (0.94–1.06)	0.99 (0.93–1.06)	0.99 (0.92–1.05)
	Education level						
	Compulsory school	1	1	1	1	1	
	Secondary school or University	5.06 (1.36–18.74)	3.48 (1.02–11.90)	2.74 (0.74–10.08)	3.98 (0.98–16.11)	2.94 (0.77–11.29)	2.82 (0.66–12.03)
	Sick leave diagnosis						
	Pain diagnoses	1	1	1	1	1	
	mental disorder diagnoses	1.85 (0.60–5.69)	1.75 (0.62–4.97)	1.35 (0.46–3.93)	1.85 (0.63–5.41)	2.16 (0.70–6.65)	3.82 (1.21–12.01)
	Both pain and mental disorder diagnoses	0.67 (0.23–1.99)	0.54 (0.19–1.53)	0.76 (0.25–2.30)	0.95 (0.33–2.78)	1.19 (0.41–3.52)	1.05 (0.36–3.06)
	HADS^c^ Anxiety	1.12 (0.99–1.25)	1.04 (0.94–1.16)	1.02 (0.91–1.14)	1.06 (0.95–1.18)	1.04 (0.93–1.17)	1.00 (0.89–1.12)
	HADS^c^ Depression	0.90 (0.78–1.03)	0.88 (0.78-1.00)	0.93 (0.81–1.06)	0.92 (0.81–1.05)	0.89 (0.77–1.01)	0.86 (0.75–0.99)
	General self-efficacy (GSE)	1.92 (0.85–4.37)	0.94 (0.44-2.00)	1.31 (0.59–2.88)	1.43 (0.64–3.19)	1.57 (0.71–3.44)	0.87 (0.39–1.96)
	Employment contract						
	Not employed	1	1	1	1	1	
	Employed	3.17 (1.13–8.91)	2.00 (0.78–5.13)	2.56 (0.90–7.29)	2.41 (0.83–7.04)	0.91 (0.31–2.67)	0.90 (0.31–2.63)
	Extent of sick leave						
	Full-time	1	1	1	1	1	1
	Part-time	13.82 (5.13–37.26)	7.69 (3.12–18.99)	13.14 (4.26–40.57)	9.51 (3.39–26.71)	12.05 (4.06–35.74)	9.25 (3.26–26.30)
	Years with sick leave compensation	0.90 (0.78–1.04)	0.88 (0.77-1.00)	0.76 (0.65–0.90)	0.84 (0.73–0.98)	0.89 (0.76–1.03)	0.89 (0.76–1.03)
Nagelkerke r^2^ for the adjusted models for each follow-up year		0.522	0.412	0.473	0.435	0.432	0.440

Logistic regression analyses presenting the odds ratio (OR) and 95% confidence interval (95% CI) for having employment as the main source of income at follow up two to seven years from inclusion

^a^Acceptance and commitment therapy (ACT) intervention group

^b^Multidisciplinary team assessment and individualized treatment (MDT) intervention group

^c^Hospital Anxiety and Depression Scale

^d^Adjusted models = Group allocation + Age + Education level + Sick leave diagnosis + HADS + GSE + Employment contract + Extent of sick leave + Years with sick leave compensation

In the crude binary logistic regression analysis, statistically significant differences were found in the work participation outcome, favoring the ACT group over the control group at follow-up years six (OR 2.53, 95% CI 1.16–5.52) and seven (OR 2.42, 95% CI 1.08–5.40). In the adjusted logistic regression analyses, statistically significant differences were found in the work participation outcome, also favoring the ACT group over the control group at follow-up years four (adjusted OR 3.21, 95% CI 1.03–9.96), six (adjusted OR 3.73, 95% CI 1.20–11.59), and seven (adjusted OR 3.99, 95% CI 1.23–12.91). In the adjusted logistic regression models, Nagelkerke r^2^ ranged between 0.41 and 0.52. See Table 3.

Logistic regression sensitivity analyses were performed, in which non-responders were categorized as not having income from employment (reference category) in the outcome. Similar results were found in the adjusted logistic regression sensitivity analyses (Table 4). The MDT intervention had a statistically significant favorable effect on the work participation outcome at follow-up in years four (adjusted OR 2.76, 95% CI 1.31–5.79), five (adjusted OR 3.02, 95% CI 1.40–6.51), and six (adjusted OR 2.48, 95% CI 1.16–5.30), in the sensitivity analyses. The ACT intervention demonstrated a favorable and statistically significant effect on the work participation outcome at follow-up in years four (adjusted OR 2.92, 95% CI 1.24–6.88), six (adjusted OR 2.93, 95% CI 1.23–7.01), and seven (adjusted OR 2.55, 95% CI 1.10–5.93), in the sensitivity analyses.

**Table 4:** Logistic regression sensitivity analyses presenting odds ratios for having employment as the main source of income at follow-up in years two, three, four, five, six, and seven

		Two years	Three years	Four years	Five years	Six years	Seven years
		OR (95% CI)	OR (95% CI)	OR (95% CI)	OR (95% CI)	OR (95% CI)	OR (95% CI)
Crude	Group allocation						
	Control	1	1	1	1	1	1
	ACT intervention^a^	1.13 (0.62–2.08)	1.15 (0.63–2.08)	1.63 (0.87–3.04)	1.54 (0.79–3.01)	1.91 (1.01–3.61)	1.69 (0.91–3.14)
	MDT intervention^b^	1.24 (0.73–2.11)	1.10 (0.65–1.87)	1.53 (0.87–2.69)	1.77 (0.98–3.20)	1.55 (0.86–2.77)	1.32 (0.76–2.31)
Adjusted models^d^	Group allocation						
	Control	1	1	1	1	1	1
	ACT intervention^a^	1.83 (0.77–4.36)	1.81 (0.79–4.12)	2.92 (1.24–6.88)	2.20 (0.90–5.36)	2.93 (1.23–7.01)	2.55 (1.10–5.93)
	MDT intervention^b^	1.76 (0.84–3.65)	1.80 (0.89–3.61)	2.76 (1.31–5.79)	3.02 (1.40–6.51)	2.48 (1.16–5.30)	2.08 (1.00-4.313)
	Age	1.06 (1.02–1.11)	1.04 (1.00-1.08)	1.02 (0.98–1.07)	1.02 (0.97–1.06)	1.01 (0.97–1.06)	1.00 (0.96–1.05)
	Education level						
	Compulsory school	1	1	1	1	1	1
	Secondary school or University	5.43 (1.94–15.17)	4.28 (1.63–11.20)	3.90 (1.40-10.35)	5.00 (1.59–15.73)	3.17 (1.35–11.32)	4.75 (1.52–14.82)
	Sick leave diagnosis						
	Pain diagnoses	1	1	1	1	1	1
	Mental disorder diagnoses	1.03 (0.46–2.29)	1.12 (0.53–2.39)	1.04 (0.48–2.26)	1.21 (0.55–2.70)	1.25 (0.56–2.81)	1.73 (0.80–3.77)
	Both pain and mental disorder diagnoses	0.59 (0.27–1.33)	0.52 (0.24–1.15)	0.68 (0.30–1.51)	0.79 (0.346-1.80)	0.96 (0.43–2.16)	0.96 (0.43–2.13)
	HADS^c^ Anxiety	1.09 (1.00-1.19)	1.04 (0.95–1.13)	1.01 (0.93–1.11)	1.02 (0.94–1.12)	1.03 (0.94–1.12)	0.99 (0.91–1.08)
	HADS^c^ Depression	0.90 (0.81–0.995)	0.89 (0.81–0.99)	0.93 (0.84–1.03)	0.93 (0.83–1.03)	0.92 (0.83–1.02)	0.92 (0.83–1.02)
	General self-efficacy (GSE)	1.47 (0.76–2.84)	0.95 (0.51–1.78)	1.14 (0.61–2.14)	1.12 (0.58–2.17)	1.38 (0.73–2.62)	0.99 (0.53–1.85)
	Employment contract						
	Not employed	1	1	1	1	1	1
	Employed	2.08 (0.85–5.11)	1.67 (0.73–3.82)	1.91 (0.79–4.59)	1.86 (0.73–4.74)	0.97 (0.40–2.33)	1.05 (0.45–2.43)
	Extent of sick leave						
	Full-time	1	1	1	1	1	1
	Part-time	6.43 (2.97–13.93)	4.15 (2.04–8.43)	4.34 (2.08–9.04)	4.59 (2.11–10.01)	5.62 (2.53–12.47)	4.39 (2.08–9.28)
	Years with sick leave compensation	0.99 (0.90–1.10)	0.96 (0.87–1.06)	0.92 (0.83–1.01)	0.96 (0.86–1.06)	0.99 (0.89–1.09)	0.97 (0.88–1.08)
Nagelkerke r^2^ for the adjusted models for each follow-up year		0.391	0.304	0.314	0.305	0.300	0.292

A worst-case scenario was analysis was carried out as a sensitivity analyses, where nonresponses (*n* = 182) to the follow-up question (and thereby missing data in the outcome) were assumed to reflect the “worst” outcome; in this analyses, that meant not having employment as the main source of income

Odds ratio (OR), 95% CI: 95% confidence interval for having employment as the main source of income

^a^Acceptance and commitment therapy (ACT) intervention group, ^b^Multidisciplinary team assessment and individualized treatment (MDT) intervention group, ^c^Hospital Anxiety and Depression Scale

^d^Adjusted models = Group allocation + Age + Education level + Sick leave diagnosis + HADS + GSE + Employment contract + Extent of sick leave + Years with sick leave compensation

## Discussion

This study aimed to follow up on a three-arm intervention study for RTW [14–16], carried out between 2010 and 2012, with mainly female participants on long-term sick leave due to mental disorders and/or chronic pain. The objective was to assess and demonstrate work participation outcomes following a multidisciplinary team assessment and individualized treatment (MDT) intervention and a unimodal psychological treatment with an acceptance and commitment therapy (ACT) intervention, during a follow-up period of two to seven years. The results indicate that a larger proportion of participants in the MDT and ACT groups had employment as their main income source at follow-up in years four to seven, compared to the control group. Review studies that have synthesized results from multidisciplinary and multi-domain interventions conclude that multi-domain interventions seem to be effective for RTW outcomes [14, 17, 36, 37]. However, contradictory studies exist; one observational register study found that multimodal rehabilitation appears not to reduce sickness absence when implemented nationwide in the Swedish healthcare [38]. ACT promotes behavior change rather than symptom reduction, and has been suggested by researchers to be relevant for RTW [39]. Results regarding ACT interventions for people on sick leave are limited when considering RTW outcomes, and that sole ACT seems to be less effective than multimodal occupational rehabilitation interventions among individuals with musculoskeletal and common mental disorders [40].

Previous long-term studies regarding RTW outcomes that evaluate multidisciplinary rehabilitation for participants with pain related disorders have reported positive results at 3- year follow-up [41], 4.5 years after the program ended [42], and even 10 years after participation [43]. Previous long-term follow-up studies on RTW outcomes for participants with common mental disorders receiving a cognitive behavioral treatment (CBT) intervention have shown statistically significant differences in a subgroup of participants that was most at risk of permanent work exclusion, but they found no differences in the total study population [44]. Other CBT/ACT intervention studies have not reported any long-term effects on RTW outcomes in patients with mental disorders and pain [45–47]. The results in this study indicate some time-lag effect, as no major differences were seen between the intervention groups and the control group at follow-up in years two and three regarding the work participation outcome. A pattern of initial delay followed by an increased effect on RTW has been seen in a previous study regarding rehabilitation for sick-listed individuals [48]. Such time-lag effect in vocational rehabilitation projects may reflect that vulnerable groups trying to enter the workforce need time to benefit from RTW interventions [49]. The study population in this study had relatively long sick leave periods when being included, and longer periods of sick leave and absence from the labor market are known risk factors for failure to RTW [7, 8].

Nagelkerke r^2^ in the adjusted logistic regression models was between 0.41 and 0.52, depending on the follow-up year. This indicates that there are other factors beyond the intervention programs and the included variables that affected work participation over the follow-up years [50]. Although sick leave is warranted by an inability to work, it can have negative consequences and contribute to e.g., depression, unhealthy behaviors, and stress [51]. Previous research have identified several important factors for RTW [9], and different factors on a systemic/organizational level may influence the outcome, such as the current demands in the labor market [52]. It is reasonable to assume that the labor market changed during the follow-up period in several aspects, such as work content and demands, etc. Therefore, a long-term follow-up study may also reflect changes in labor market demands that influence RTW outcomes; however, these labor market conditions would have affected both the intervention groups and the control group alike.

The major findings in this study imply that participants in the MDT and ACT intervention groups were, to a larger extent, in paid work four to seven years from inclusion, compared to the control group. The findings add to the body of evidence that vocational rehabilitation may increase RTW outcomes over the long-term, among sick-listed individuals with mental disorders and/or chronic pain. There is a need for further investigation into the effectiveness of multidisciplinary RTW and ACT interventions over longer periods. In addition, qualitative research may be an alternative strategy for increasing the knowledge of the mechanisms and other factors that influence work-related outcomes.

### Strengths and limitations

The strengths of this study include its randomized controlled experimental design, the long-term follow-up period, and the use of registry data for the outcome measure. As the outcome measures depict actual income from work, this study goes beyond those that rely on sick leave compensation data, which only indirectly measures RTW.

However, this study also has some limitations. As the majority of participants were females, the results should be interpreted with caution due to the skewed gender distribution. In addition, education level, HADS, and GSE were measured after randomization (but before the interventions began), which could have influenced the scores due to knowledge of intervention group affiliation. Another limitation is that the study combines both individual treatment and organizational collaboration, which raises the question of which components mediate the effect, or if it is a combination of these factors. The reason for including organizational collaboration was to set and mutually agree on each individual’s RTW goal.

In this study, several statistical tests were carried out, raising the possibility of multiple comparison problems. Therefore, particular *p*-values should be interpreted with caution, and results should be viewed in the context of overall demonstrated trends. Several participants did not complete the long-term follow-up questionnaires that provided us with permission to retrieve registry data, which weakens the ability to make causal inferences. Dropout analysis revealed differences between the dropouts and participants who completed the long-term follow-up in: education level, HADS, GSE, and extent of sick leave. The analysis indicates that dropouts might represent a subgroup with a lower likelihood of returning to employment after a period of sick leave. Therefore, sensitivity analyses were conducted to test the robustness of the results. In these analyses, dropouts were assumed to reflect the “worst” outcome, meaning they were not considered to have employment as their main source of income. However, the results of this study should be interpreted with caution due to the proportion of missing data in the outcome measure.

## Conclusions

This long-term follow-up study of vocational rehabilitation mainly involved female participants on sick leave due to mental disorders and/or chronic pain. The study found increased work participation among participants in the MDT and ACT groups at follow-up in years four, five, six, and seven after inclusion when compared to the controls. The results over the follow-up period demonstrate some time-lag effect of the interventions, which indicates that the benefits from the MDT and ACT interventions do not materialize rapidly for individuals on long-term sick leave regarding RTW.

The study implies that it is possible to strengthen long-term work participation by using interventions that include MDT and ACT among individuals on long-term sick leave due to mental disorders and/or chronic pain. However, the findings should be interpreted with caution due to limitations, which include the skewed gender distribution and the proportion of dropouts.

## Data Availability

The datasets generated and/or analysed during the current study are not publicly available due confidentiality and ethical reasons.

## Abbreviations

ACT: Acceptance and commitment therapy
CBT: Cognitive behavioral therapy
GSE: General Self-Efficacy scale
HADS: Hospital Anxiety and Depression Scale
ITT: Intention-to-treat
LISA: Longitudinal Integration Database for Health Insurance and Labor Market Studies
MDT: Multidisciplinary team assessment and individualized treatment
RTW: Return-to-work
SPES: Swedish Public Employment Service
SPSS: Statistical Package for the Social Sciences
SSIA: Swedish Social Insurance Agency
